# Planar Phase-Variation Microwave Sensors for Material Characterization: A Review and Comparison of Various Approaches

**DOI:** 10.3390/s21041542

**Published:** 2021-02-23

**Authors:** Jonathan Muñoz-Enano, Jan Coromina, Paris Vélez, Lijuan Su, Marta Gil, Pau Casacuberta, Ferran Martín

**Affiliations:** 1 CIMITEC, Departament d’Enginyeria Electrònica, Universitat Autònoma de Barcelona, 08193 Bellaterra, Spain; Jan.Coromina@uab.cat (J.C.); Paris.Velez@uab.cat (P.V.); Lijuan.Su@uab.cat (L.S.); pau.casacubertao@e-campus.uab.cat (P.C.); Ferran.Martin@uab.cat (F.M.); 2 Departamento Ingeniería Audiovisual y Comunicaciones, Universidad Politécnica de Madrid, 28031 Madrid, Spain; marta.gil.barba@upm.es

**Keywords:** microwave sensors, stepped-impedance transmission lines, slow-wave transmission lines, meander lines, dielectric constant sensor, phase-variation sensors

## Abstract

Planar phase-variation microwave sensors have attracted increasing interest in recent years since they combine the advantages of planar technology (including low cost, low profile, and sensor integration with the associated circuitry for post-processing and communication purposes, among others) and the possibility of operation at a single frequency (thereby reducing the costs of the associated electronics). This paper reviews and compares three different strategies for sensitivity improvement in such phase-variation sensors (devoted to material characterization). The considered approaches include line elongation (through meandering), dispersion engineering (by considering slow-wave artificial transmission lines), and reflective-mode sensors based on step-impedance open-ended lines. It is shown that unprecedented sensitivities compatible with small sensing regions are achievable with the latter approach.

## 1. Introduction

Planar microwave sensors are very good candidates for material characterization and analysis. The reason is the high sensitivity of microwaves to the properties of the materials to which they interact, combined with the advantageous aspects of planar technology (including low cost and profile, compatibility with other technologies, such as microfluidics, full integration with the associated electronics, or the possibility of implementing the sensors in conformal, flexible and organic/compostable substrates, among others). 

Sensor cost, design simplicity, robustness, and performance (mainly, although not exclusively, the sensitivity) are the main aspects that determine the interest and quality of a sensing device. As mentioned before, planar sensors are typically cheap, at least as compared to bulk sensors (e.g., cavity sensors). However, the real cost of a sensor is not merely determined by the sensitive part of the device, but also by the associated electronics needed for sensor feeding, post-processing and, eventually, communication purposes. Probably, the most extended approach for sensing using microwaves is frequency variation [1,2,3,4,5,6,7,8,9,10,11,12,13,14,15,16,17,18,19,20,21,22,23,24], where, typically, a planar resonant element combined with a transmission line constitutes the sensing (microwave) part of the sensor. Frequency-variation sensors have been applied to the determination of the complex dielectric constant of solids and liquids [3,5,6,7,10,17,18,19,20,21,22,23,24], from the measurement of the resonance frequency and magnitude variation caused by the so-called material under test (MUT). Permittivity sensors based on frequency splitting have been also recently reported [25,26,27,28,29,30,31,32,33,34,35]. These sensors consist of a transmission line structure loaded with a pair of identical resonant elements, and sensing is based on resonance splitting caused by an asymmetric dielectric loading between the reference (REF) resonator and the MUT resonator. Similar to differential-mode sensors [35,36,37,38,39,40,41,42,43,44,45,46], frequency-splitting sensors are robust against cross sensitivities caused by environmental factors (e.g., changes in temperature or humidity). This is the main advantage of frequency-splitting over frequency-variation sensors. However, both sensor types need wideband interrogation signals for measurement purposes, at least covering the output dynamic range, and this represents a penalty in terms of the costs related to the associated electronics. Note that, despite the fact that vector network analyzers (VNAs) are typically used for demonstration purposes, the interrogation signal sweeping in a real scenario (where portable devices are usually a need) should be generated by means of wideband voltage-controlled oscillators (VCOs). This represents a challenging issue since a single VCO may not suffice to cover the required bandwidth of the output signal. Therefore, this may represent also a burden in terms of sensor costs. 

To alleviate the previous cost limitations, single-frequency sensors (requiring a single-tone harmonic signal for sensing) represent a good solution. Coupling modulation sensors [47,48,49,50,51,52,53,54,55,56,57,58,59,60] belong to this category. In such sensors, the sensing principle is based on modulation of the coupling between a resonator (or a set of resonators) and a transmission line, caused by a relative displacement between both elements, or by an asymmetric dielectric load in the resonant element. The typical output variable in such sensors is the magnitude of the transmission coefficient at the operating frequency, which can be easily converted to a voltage magnitude by means of an envelope detector [53,59]. The main drawback of such sensors concerns the fact that magnitude measurements exhibit a limited robustness against the effects of interference and noise, at least as compared to frequency or phase measurements. According to the previous words, single-frequency phase-variation sensors, the subject of this review paper, may represent a good tradeoff between overall costs and robustness against noise and interference effects. 

The main objective of this review paper is to point out, discuss and compare different sensing strategies devoted to performance optimization (specifically sensitivity enhancement, the typical challenging aspect) in phase-variation microwave sensors [36,37,38,45,46,61,62,63,64]. Even though examples of planar phase-variation microwave sensors focused on sensing spatial variables have been reported [65,66,67], the sensors to be discussed in the present paper are mainly devoted to material characterization. Nevertheless, an example of a rotation sensor based on phase variation is also included in the paper (other sensing strategies different than phase variation are not discussed in the paper). The working principle in such sensors is the phase variation in the sensing element, a transmission line section (either conventional or artificial), caused by variations in the effective dielectric constant of the MUT. Specifically, the considered strategies for sensitivity optimization through the measurement of the phase are (i) line elongation through meandering, (ii) phase velocity reduction by means of slow-wave transmission lines, and (iii) impedance contrast in reflective-mode step-impedance open-ended lines. A phase-variation differential sensor based on highly dispersive composite right/left handed (CRLH lines), where the phase information is transformed to magnitude information, is also included in the paper.

Organization of the paper is as follows: In Section 2, the considered sensing approaches for sensitivity optimization are justified. Section 3, Section 4 and Section 5 are devoted to phase-variation sensors based on meander lines, slow-wave transmission lines, and reflective-mode step-impedance lines, respectively. Other phase-variation sensors, including a prototype devoted to measure angular displacements and a differential phase-variation sensor with phase-to-magnitude conversion, are reported in Section 6. A comparative analysis is carried out in Section 7. Finally, the main concluding remarks are highlighted in Section 8.

## 2. Towards Sensitivity Optimization in Phase-Variation Microwave Sensors

The typical performance parameters in microwave sensors are the sensitivity, the resolution, the accuracy, the input and output dynamic ranges, the linearity, and the repeatability. Although such parameters are used as indicators of the quality of a sensor, other aspects also determine the preferred sensor approach for a certain application. Such aspects are sensor cost, size, design complexity, and robustness (understood as the capacity of the sensor to mitigate the pernicious effects of electromagnetic interference, noise, cross-sensitivities, and harsh environments). Phase-variation microwave sensors are good candidates for sensing, since this type of sensors exhibits a good combination of the previous characteristics and attributes. In many applications, a high sensitivity and resolution is required. For example, highly sensitive sensors are needed in several industrial processes, including the heavy and automotive industry (e.g., defect detection in samples, material composition measurements, motion control), or the food and related industry (e.g., wine fermentation processes), among others. Other applications related to health and bio-sensing (e.g., monitoring the electrolyte content or the glucose level in blood or urine) also require high sensitivity. Thus, sensors able to detect small changes in material composition are highly demanded. Such changes typically manifest through a variation in the dielectric constant (or, more generally, the complex permittivity) of the so-called material, or sample, under test (MUT), a measurable quantity using microwave technology.

**Figure 1:**
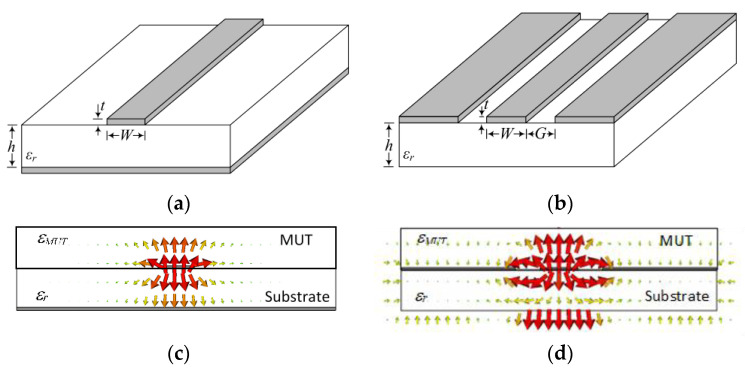
Perspective view of a microstrip line (**a**) and CPW (**b**) surrounded by air, and cross-sectional view of the microstrip line (**c**) and CPW (**d**) covered by a MUT. The electric field lines are indicated.

The phase velocity of an electromagnetic signal propagating in a certain medium depends on the dielectric constant (or relative permittivity), *ε*, of that medium, according to [68]:

(1) $$v_{p}=\frac{c}{\sqrt{\varepsilon }}\;$$

where *c* is the speed of light in vacuum. Consequently, a variation in *ε* can be detected through the variation in the phase of the wave measured at a certain point, provided the phase depends on the phase velocity. However, most phase-variation sensors are implemented by means of planar structures, specifically open transmission lines, such as microstrip lines, coplanar waveguide (CPW) transmission lines, etc., see Figure 1. In such lines, the velocity of the propagating waves (also called phase velocity) is given by:

(2) $$v_{p}=\frac{c}{\sqrt{\varepsilon_{eff}}}\;$$

where *ε_eff_* is the so-called effective dielectric constant. The effective dielectric constant depends on the dielectric constant of the substrate, *ε_r_*, and the dielectric constant of the medium surrounding the line. Such medium is typically air, at least when the lines act as distributed circuit elements. However, in transmission-line phase-variation sensors, the line (the sensing element) should be covered by the MUT. Thus, the dielectric constant of the MUT, *ε_MUT_*, determines the value of the effective dielectric constant, which is also a function of the transverse geometry of the line (the specific value of *ε_eff_* is given by the distribution of the electric field lines between the substrate and the MUT on top of the line). The specific formula is:

(3) $$\varepsilon_{eff}=\frac{\varepsilon_{r}+\varepsilon_{MUT}}{2}\;$$

for CPW transmission lines, and:

(4) $$\varepsilon_{eff}=\frac{\varepsilon_{r}+\varepsilon_{MUT}}{2}+\frac{\varepsilon_{r}-\varepsilon_{MUT}}{2}F\;$$

for microstrip lines. In (4), *F* is a geometry factor given by:

(5a) $$F={\left(1+12\frac{h}{W_{s}}\right)}^{-\frac{1}{2}}\;$$

for *W_s_*/*h* ≥ 1, or by

(5b) $$F={\left(1+12\frac{h}{W_{s}}\right)}^{-\frac{1}{2}}+0.04{\left(1-\frac{W_{s}}{h}\right)}^{2}$$

for *W_s_*/*h* < 1, where *h* and *W_s_* are the substrate thickness and the width of the sensing line, respectively, and it is assumed that *t << h*, where *t* is the thickness of the metallic layer.

In Equations (3) and (4), it is assumed that the MUT is semi-infinite in the vertical direction. The validity of Equation (3) is also examined in the case of a CPW transmission line with a semi-infinite substrate. If such conditions are not fulfilled, (3) and (4) no longer provide the effective dielectric constants for the CPW and microstrip lines, respectively. Nevertheless, the functionality of the sensor is preserved, despite the fact that such approximations are not satisfied. Namely, a change in *ε_MUT_* generates a variation in *ε_eff_*, which in turn modifies the phase velocity of the transmission line. The phase of the line (or electrical length) depends on the angular frequency, *ω*_0_, the line length *l*, and the phase velocity, *v_p_*, according to:

(6) $$\phi =\frac{\omega_{0}l}{v_{p}}=\frac{\omega_{0}l}{c}\sqrt{\varepsilon_{eff}}\;$$

Thus, sensing *ε_MUT_*, or any other variable related to it, e.g., material composition, solute content in a liquid solution, etc., is possible from the measurement of the phase of the line, such as Equation (6) suggests.

The phase of the line, *ϕ*, is not necessarily the output variable in phase-variation sensors. For example, there are sensors where the phase information is transformed to magnitude information [38,45,46]. Nevertheless, it is clear that for sensitivity optimization, it is necessary to achieve the maximum possible variation of the phase of the line with the dielectric constant of the MUT. The sensitivity in phase-variation sensors where the output variable is the phase (those of the foremost interest in the present study) is thus defined as:

(7) $$S=\frac{d\phi }{d\varepsilon_{MUT}}\;$$

In ordinary lines, the following result is obtained:

(8) $$S=\frac{d\phi }{d\varepsilon_{MUT}}=\frac{d\phi }{d\varepsilon_{eff}}\cdot \frac{d\varepsilon_{eff}}{d\varepsilon_{MUT}}=\frac{\omega_{0}l}{2c\sqrt{\varepsilon_{eff}}}\cdot \frac{d\varepsilon_{eff}}{d\varepsilon_{MUT}}\;$$

Using Equation (3), the sensitivity for a CPW line is found to be:

(9) $$S=\frac{\omega_{0}l}{2\sqrt{2}c\sqrt{\varepsilon_{r}+\varepsilon_{MUT}}}\;$$

whereas for microstrip lines, the sensitivity is inferred by introducing Equation (4) in (8), i.e.,:

(10) $$S=\frac{\omega_{0}l}{2\sqrt{2}c\sqrt{\varepsilon_{r}\frac{1+F}{{\left(1-F\right)}^{2}}+\varepsilon_{MUT}\frac{1}{1-F}}}\;$$

It is interesting to mention that for a CPW sensing line with thick enough MUT and substrate, *S* does not depend on the transverse geometry, contrarily to microstrip lines (note the dependence of *S* on *F* in (10)). In both cases, reducing the dielectric constant of the substrate, *ε_r_*, boosts up the sensitivity. However, the dependence of the sensitivity with *ε_r_* is through the square root, and hence, it is limited. Moreover, aspects such as the compatibility with the associated sensor electronics, or cost, may dictate the sensor substrate. Thus, *ε_r_* cannot be considered a true design parameter, useful for sensitivity optimization. Inspection of (9) and (10) reveals that the sensitivity is proportional to the product *ω*_0_*l*. Therefore, it is clear that, for sensitivity optimization, sensor operation at high frequencies and/or the use of long sensing lines are good strategies for sensitivity optimization. However, operating at high frequencies is not exempt of certain complexity in the generation of the harmonic signal required for sensing. Therefore, the typical approach for sensitivity enhancement in phase-variation sensors based on transmission lines is line meandering. By meandering, elongated lines with reasonable shape factors for the sensing region, delimited by the region to which the meandered line is circumscribed, are obtained. An example of a phase-variation sensor devoted to dielectric constant measurements, operating in differential mode, and based on a pair of meandered lines is reported in [37], and will be the subject of Section 3.

A different strategy for sensitivity optimization in phase-variation sensors exploits dispersion engineering in artificial transmission lines [69,70,71]. The idea behind this approach is to reduce the phase velocity of the (artificial) sensing line. For that purpose, composite right/left-handed (CRLH) lines, first reported in [72], electro-inductive wave transmission lines [69,73], and slow-wave transmission lines [69,74,75,76] are useful. In an ordinary line, the phase velocity can only be reduced by increasing the dielectric constant of the substrate, *ε_r_*. However, the net effect of increasing *ε_r_* is a reduction in the sensitivity, as it can be appreciated in Equations (9) and (10). Let us clarify this aspect. Note that from Equation (6), the sensitivity can be alternatively expressed as:

(11) $$S=\frac{d\phi }{d\varepsilon_{MUT}}=\frac{d\phi }{dv_{p}}\cdot \frac{dv_{p}}{d\varepsilon_{MUT}}=-\frac{\omega_{0}l}{v_{p}^{2}}\cdot \frac{dv_{p}}{d\varepsilon_{MUT}}\;$$

and, apparently, a reduction of *v_p_* increases the sensitivity. However, in an ordinary transmission line, the last term in Equation (11) can be expressed as:

(12) $$\frac{dv_{p}}{d\varepsilon_{MUT}}=\;\frac{dv_{p}}{d\varepsilon_{eff}}\cdot \frac{d\varepsilon_{eff}}{d\varepsilon_{MUT}}=-\frac{v_{p}^{3}}{2c^{2}}\cdot \frac{d\varepsilon_{eff}}{d\varepsilon_{MUT}}\;$$

and it is obvious that, by introducing Equation (12) into Equation (11), the sensitivity, in terms of *v_p_*, is found to be:

(13) $$S=\frac{d\phi }{d\varepsilon_{MUT}}=\frac{d\phi }{dv_{p}}\cdot \frac{dv_{p}}{d\varepsilon_{MUT}}=\frac{\omega_{0}lv_{p}}{2c^{2}}\cdot \frac{d\varepsilon_{eff}}{d\varepsilon_{MUT}}\;$$

and it increases with *v_p_*, i.e., it decreases with *ε_r_*. However, in artificial lines, the phase velocity can be reduced by different approaches, and this opens the possibility to enhance the sensitivity based on the reduction of the phase velocity.

The last phase-variation sensors studied in this review paper are one-port reflective-mode sensors based on either half- or a quarter-wavelength open-ended transmission line resonators. In such sensors the output variable is the phase of the reflection coefficient, *ϕ_ρ_*. As it will be shown, the sensitivity to the dielectric constant of the MUT can be enhanced by appropriately choosing the characteristic impedance of the half- or quarter-wavelength open-ended sensing lines. However, as it has been demonstrated [62], the sensitivity can be unprecedentedly enhanced by cascading high/low impedance quarter-wavelength line sections between the sensing line and the input port of the structure. There is a multiplicative effect in the sensitivity, controlled by the impedance contrast of the quarter-wavelength line sections. The main benefit of these sensors, based on a step-impedance transmission line configuration, is the fact that the sensitivity can be enhanced without the need to increase the dimensions of the sensing region. Therefore, sensors with huge sensitivity compatible with small sensing areas can be designed and fabricated. Indeed, a figure of merit in phase-variation sensors is the ratio between the maximum sensitivity, *S*, and the sensing area, *A*, expressed in terms of the squared wavelength, i.e.,:

(14) $$FoM=\frac{S}{A}\;$$

As will be shown in Section 5, these reflective-mode phase-variation sensors exhibit a very high *FoM*, and are therefore excellent candidates for applications requiring high sensitivity and resolution, as well as small MUT samples.

## 3. Phase-Variation Sensors Based on Meandered Lines

As mentioned before, sensitivity optimization in phase-variation sensors implemented with sensitive regions based on meandered lines relies on the elongated length of the sensitive lines. As an example of the potential of meandered lines for sensitivity optimization, let us consider a differential sensor implemented with two identical meandered lines [37]. The sensing principle is the variation of the electrical length of the sensing line, as compared to the reference line, when such lines are loaded with the MUT and REF samples, respectively. The input and output variables of the sensor are the differential dielectric constant, Δ*ε* = *ε_REF_* − *ε_MUT_*, and the differential phase of the lines, Δ*ϕ* = *ϕ_REF_* − *ϕ_MUT_*, respectively, where *ε_REF_* and *ε_MUT_* are the dielectric constants of the REF and MUT samples, respectively, and *ϕ_REF_* and *ϕ_MUT_* are the electrical lengths of the REF and MUT line, respectively. In order to express Δ*ϕ* in terms of Δ*ε*, let us first write the differential phase as a function of the effective dielectric constants of the REF line, *ε_eff,REF_*, and MUT line, *ε_eff,MUT_* , i.e.,:

(15) $$∆\phi =\beta_{0}l\left(\sqrt{\varepsilon_{eff,REF}}-\sqrt{\varepsilon_{eff,MUT}}\right)\;$$

where *β*_0_ = *ω*_0_/*c* is the phase constant in vacuum. These effective dielectric constants are given by (3) or by (4), depending on the considered line technology (microstrip or CPW). Obviously, the sub-index MUT in (3) or (4) should be replaced with the sub-index REF for the corresponding line. Introducing the effective dielectric constants of both lines in (15), the following result is obtained:

(16) $$∆\phi =\frac{\beta_{0}l}{\sqrt{2}}\left(\sqrt{\varepsilon_{r}\left(1+F\right)+\varepsilon_{REF}\left(1-F\right)}-\sqrt{\varepsilon_{r}\left(1+F\right)+\varepsilon_{MUT}\left(1-F\right)}\right)\;$$

where line in implementation in microstrip technology has been considered. 

Let us now consider that the interest is to measure small variations of the dielectric constant of the MUT in reference to the one of the REF material, with a maximum input dynamic range designated as ±Δ*ε_max_*. Under these conditions, it follows that Δ*ε* << *ε_REF_*, and Equation (16) can be approximated by:

(17) $$∆\phi =\frac{\beta_{0}l}{2\sqrt{2}}\frac{∆\varepsilon \left(1-F\right)}{\sqrt{\varepsilon_{r}\left(1+F\right)+\varepsilon_{REF}\left(1-F\right)}\;}\;$$

For sensitivity optimization, and taking into account the considered maximum input dynamic range, it is convenient to force Δ*ϕ* = ±π when Δ*ε* = ±Δ*ε_max_*. Introducing these extreme values in Equation (17) and isolating the length of the lines, one obtains:

(18) $$l=2\sqrt{2}\pi \frac{\sqrt{\varepsilon_{r}\left(1+F\right)+\varepsilon_{REF}\left(1-F\right)}}{\beta_{0}∆\varepsilon_{max}\left(1-F\right)}\;$$

and the sensitivity is:

(19) $$S=\frac{\pi }{∆\varepsilon_{max}}=\frac{\beta_{0}l}{2\sqrt{2}}\cdot \frac{\left(1-F\right)}{\sqrt{\varepsilon_{r}\left(1+F\right)+\varepsilon_{REF}\left(1-F\right)}\;}\;$$

with *l* given by Equation (18). Note that by simply increasing *l*, the sensitivity can be made as high as desired (with the operating frequency, and consequently *β*_0_, set to a certain value). In view of Equation (18), it is clear that small dielectric constant substrates help to reduce the length of the lines (by decreasing *ε_REF_*, the same effect is obtained, but this parameter is not a design variable).

For the prototype device sensor, reported in [37], a Rogers RO4003C substrate with dielectric constant *ε_r_* = 3.5, thickness *h* = 0.8128 mm and loss tangent tanδ*_SUB_* = 0.0027 was used. A piece of Nelco N4350‑13RF substrate, with dielectric constant *ε_REF_* = 3.55, and dissipation factor tanδ*_REF_* = 0.0065, was considered to be the REF sample. With such thickness of the REF sample, it can be considered to be semi-infinite in the vertical direction. With these material properties, the width of the REF line necessary to provide a 50 Ω characteristic impedance was found to be *W_s_* = 1.6 mm. The considered input dynamic range measurement was limited to Δ*ε_max_* = 0.45. Such dynamic range corresponds to 12.7% variations of the dielectric constant of the REF sample. By evaluating (18), it follows that *β*_0_*l* = 84.23 rad. This gives a length for the meandered lines of *l* = 670.3 mm, by considering an operating frequency of *f*_0_ = *ω*_0_/2π = 6 GHz. Such long line length is the motivation for line meandering. Nevertheless, similar results are achievable by means of straight lines (but in this case with the penalty of an excessive sensor dimension in the direction of the line axis).

**Figure 2:**
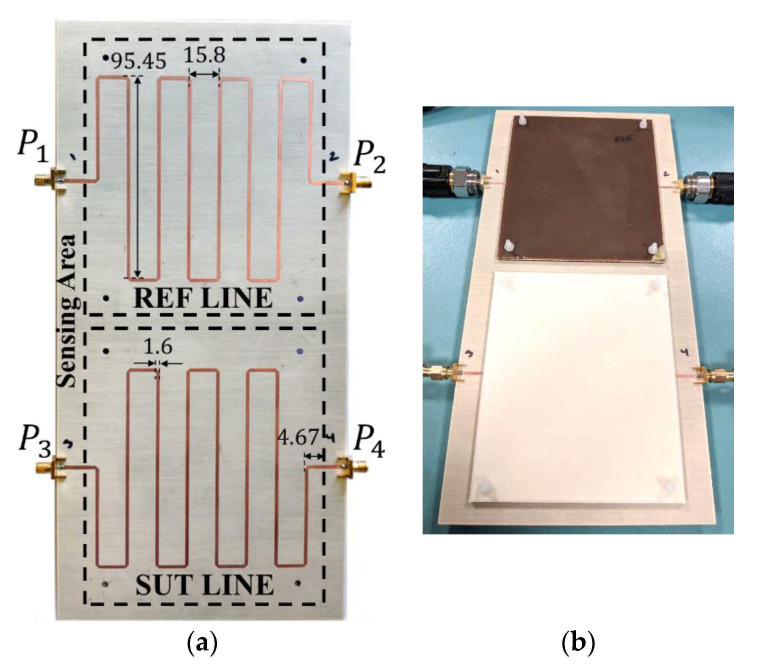
Photograph of the differential-mode phase variation sensor based on a pair of meandered lines, without (**a**) and with (**b**) REF and MUT (or SUT) samples on top of it (dimensions are given in mm). Reprinted with permission from [37].

**Figure 3:**
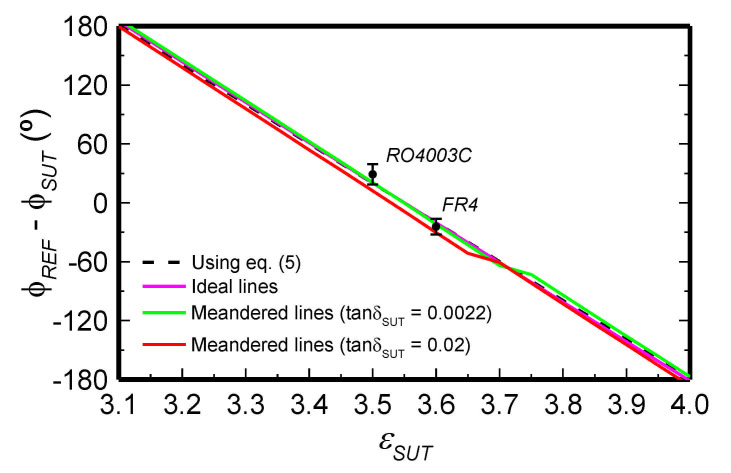
Simulated differential phase at *f*_0_ = 6 GHz for the sensor of Figure 2, as a function of the dielectric constant of the MUT, parameterized by the loss tangent of the MUT. The curve given by Equation (17), or by Equation (5) in the source paper [37], is also included. The mean of the measured differential phases (with *N* = 3 independent measurements) for the indicated materials, as well as the error bars inferred from the standard deviation, are also included. Reprinted with permission from [37].

A photograph of the fabricated bare sensor, with relevant dimensions indicated, is depicted in Figure 2a, whereas Figure 2b shows the sensor loaded with the REF and MUT samples. The differential phase of the structure (inferred from full-wave electromagnetic simulation using Keysight Momentum) as a function of the dielectric constant of the MUT (or sample under test, SUT), by considering variations in the vicinity of *ε_REF_* = 3.55 with Δ*ε_max_* = 0.45, is depicted in Figure 3. In reality, Figure 3 shows the results obtained by considering different values of the loss tangent of the MUT. The fact that the curves are very similar indicates that the loss factor of the MUT does not play an important role on the differential phase. The simulated curves of Figure 3 are in very good agreement with the analytical Equation (17) (Equation (5) in the source paper [37]), also included in the figure. Such agreement validates the previous analysis, and points out that it is not necessary to know in advance the loss tangent of the MUT sample in order to determine its dielectric constant. Note that for the MUT sample with the highest or lowest dielectric constant (corresponding to ±Δ*ε_max_*), the differential phase is found to be approximately Δ*ϕ* = *ϕ_REF_* − *ϕ_MUT_ =* ±π. The sensitivity is roughly constant, in agreement with Equation (19), and it is found to be *S* = *d*Δ*ϕ*/*d*Δ*ε* = −415.6° (very close to the theoretical value, i.e., −400°). With such high sensitivity, the sensor is able to detect extremely small differential dielectric constants.

The sensor was experimentally validated in [37]. For that purpose, the sensing region was first loaded with a commercial (uncladded) microwave substrate with well-known dielectric constant, i.e., the *FR4* substrate with *ε_MUT_* = 4.5. The thicknesses of the REF and MUT samples are 3.3 mm and 3.2 mm, respectively (such thicknesses were achieved by stacking two samples). With these thicknesses, the samples can be roughly considered semi-infinite. It is important to indicate that *ε_MUT_* (or the corresponding differential dielectric constant Δ*ε*) is out of the considered input dynamic range. Nevertheless, the measured differential phase, Δ*ϕ*, is a periodic function with Δ*ε* (actually, Δ*ϕ* varies linearly with Δ*ε*, but any measured value of Δ*ϕ* out of the range [−π,+π] is expressed by its equivalent value within that range). Thus, it is possible to check the validity of the approach from the measured differential phase (the limitation is dictated by the requirement Δ*ε* << *ε_REF_*). According to the results of Figure 3, the expected value of the differential phase for *ε_MUT_* = 4.5 is Δ*ϕ* = −19.18° (equivalent to *ε_MUT_* = 3.6), whereas the mean of the measured values at *f*_0_ = 6 GHz (*N* = 3 independent measurement were carried out) is Δ*ϕ* = −24.23°. These phases are in very good agreement, taking into account the relatively large value of *ε_MUT_*. Another experiment was also carried out by using an MUT consisting of an uncoated piece of the Rogers RO4003C substrate with *ε_MUT_* = 3.5 (satisfying Δ*ε* << *ε_REF_*) and thickness 3.05 mm (also achieved by stacking two samples). The resulting mean of the measured differential phases at *f*_0_ = 6 GHz (also with *N* = 3 independent measurements) is Δ*ϕ* = 28.97°, in good agreement with the expected value (Δ*ϕ* = 20.34°). Figure 3 includes also the measured differential phases at *f*_0_ = 6GHz, in order to ease the comparison with the theoretical value. Note that from the measured phases, the dielectric constant values inferred from Equation (17) for *FR4* and *RO4003C* are found to be 4.51 and 3.48, respectively, very close to the nominal values. In order to minimize the effects of the air gap (between the substrate and the REF and MUT samples), the MUT and REF samples were pressed against the substrate. For that purpose, Teflon screws (depicted in Figure 2b) were used.

**Figure 4:**
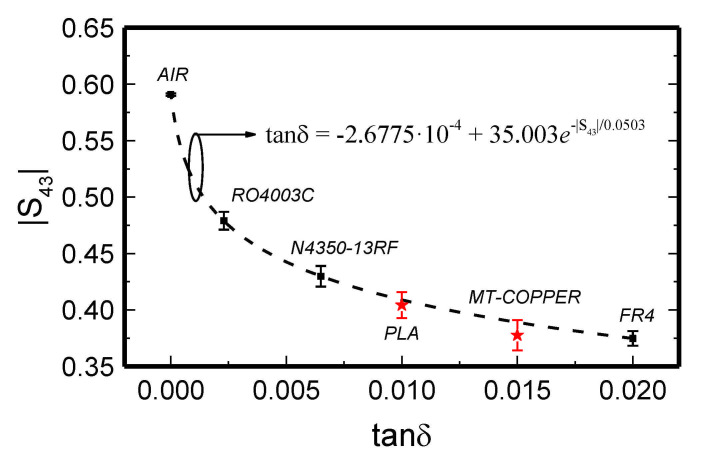
Measured transmission coefficient at *f*_0_ = 6 GHz for different (indicated) MUT samples. The mean values, as well as the error bars, obtained from *N* = 3 independent measurements are depicted. Reprinted with permission from [37].

As it was indicated in [37], the sensor of Figure 2 can also be applied to estimate the loss tangent of the MUT. However, such measurement is based on a single-ended measurement, namely, the magnitude of the transmission coefficient of the sensing line, |*S*_43_|.The simulated |*S*_43_| for different values of the loss tangent of the MUT was obtained as a function of the dielectric constant of the MUT through full-wave electromagnetic simulation and reported in [37]. The results indicate that the dependence of |*S*_43_| on *ε_MUT_* is very soft. Nevertheless, the access lines and connectors (not accounted for in the simulations) generate inevitable discrepancies between the simulated and measured transmission coefficient. Therefore, in [37] it was measured the magnitude of the transmission coefficient for several samples with different value of the loss tangent. The values of |*S*_43_| obtained at *f*_0_ are depicted in Figure 4. A calibration curve can be generated using these values (see Figure 4), and this curve is useful to determine the loss tangent of the MUT from the measured value of |*S*_43_|. The FR4 and Rogers 4003C substrates indicated in the previous paragraph, as well as the Nelco N4350‑13RF substrate (the REF sample for the differential phase measurements), were used for the generation of the calibration curve. Nevertheless, the transmission coefficient of the unloaded MUT line (corresponding to tanδ_SUT_ = 0) was measured, as it provides an additional data point. 

To validate the approach for the determination of the loss tangent, two samples were fabricated with a 3D-printer (model Ultimaker 3 Extended). The loss factor of such samples, measured by means of a Keysight 85072A commercial resonant cavity were found to be tanδ_SUT_ = 0.010 (for MUT1, fabricated by considering *PLA* as filament) and tanδ_SUT_ = 0.016 (for MUT2, fabricated by considering RS Pro MT‑COPPER as filament). The measured values of the insertion loss at *f*_0_ that are obtained by loading the MUT line with these 3D-printed samples are indicated in Figure 4. It can be seen that the resulting points are in reasonable agreement with the calibration curve. Therefore, it is demonstrated that such curve can be used to estimate the loss tangent of the MUT sample.

## 4. Phase-Variation Sensors Based on Slow-Wave Artificial Transmission Lines

**Figure 5:**
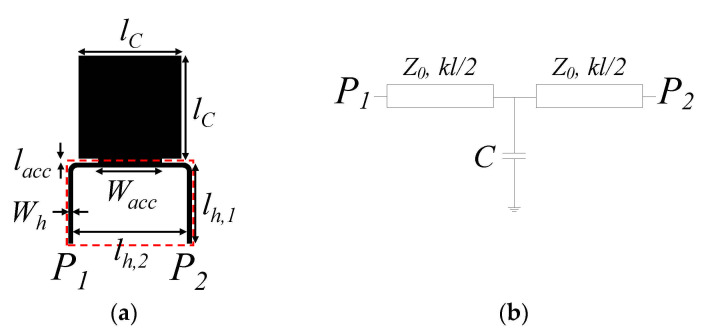
Typical topology (**a**) and equivalent circuit schematic (**b**) of the unit cell of a capacitively-loaded slow-wave transmission line. Relevant dimensions are indicated.

Let us now review a different approach for sensitivity enhancement in phase-variation microwave sensors, consisting in implementing the sensing region with a slow-wave artificial transmission line [69]. These lines exhibit a phase velocity smaller than that of ordinary lines. To achieve such slow-wave effect, several strategies are possible, including line loading with a periodic array of shunt connected capacitors [76,77,78,79,80,81,82,83], series inductors [84,85,86,87], or both elements simultaneously [88,89]. In all these cases of reactive loading, the effective capacitance and/or the effective inductance of the line are increased, and the result is a decrease in the phase velocity. To illustrate the approach, a slow-wave transmission line-based sensor implemented by means of a capacitively-loaded line, first reported in [61], is presented. The topology and circuit schematic (unit cell) of these periodically loaded lines consists of a host line loaded with a shunt capacitor *C* at its central position (Figure 5). The electrical length of the host line at the design (operating) frequency (*f*_0_ = *ω*_0_/2π) and the characteristic impedance are designated by *θ* = *kl* and *Z*_0_, respectively (*k* is the phase constant of the host line and *l* its length). From the transfer matrix approach, the electrical length, *ϕ* = *βl*, of the unit cell of this capacitively-loaded line (*β* being the phase constant of the unit cell) and the characteristic (or Bloch) impedance *Z_B_*, are found to be [69,82]

(20) $$\cos\phi =\cos\theta -\frac{\omega_{0}CZ_{0}}{2}\sin\theta \;$$

(21) $$Z_{B}=Z_{0}\frac{\sin\theta -\omega_{0}CZ_{0}{\left(\sin\theta /2\right)}^{2}}{\sin\phi }\;$$

The usual design (input) parameters of the slow-wave transmission line of Figure 5 are *ϕ* and *Z*_B_ (typically dictated by circuit specifications [82]). However, from the assigned values to these parameters, it is not possible to univocally determine *θ*, *Z*_0_ and *C* from (20) and (21). An additional (third) condition is required for that purpose, and such condition is usually the so-called slow wave ratio, or ratio between the phase velocities of the slow-wave transmission line and ordinary (host) line, given by:

(22) $$swr=\frac{v_{p}}{v_{p0}}=\;\frac{\omega /\beta }{\omega /k}=\frac{\theta }{\phi }\;$$

Let us consider that the sensing region includes the host line (such region is indicated by the dashed rectangle in Figure 5), but not the patch (shunt-connected) capacitors. It is expected that a variation in the dielectric constant of the MUT, placed on top of the sensing area, modifies the phase velocity of the host line, *v_p_*_0_, as well as its characteristic impedance, *Z*_0_. Thus, the derivative of the phase of the reactively loaded line, *ϕ*, with respect to *ε_MUT_*, the sensitivity, can be expressed as:

(23) $$S=\frac{d\phi }{d\varepsilon_{MUT}}=\frac{d\phi }{d\theta }\cdot \frac{d\phi }{dv_{p0}}\cdot \frac{dv_{p0}}{d\varepsilon_{MUT}}+\frac{d\phi }{dZ_{0}}\cdot \frac{dZ_{0}}{d\varepsilon_{MUT}}\;$$

with:

(24a) $$\frac{d\phi }{d\theta }=\frac{\sin\theta +\frac{\omega_{0}CZ_{0}}{2}\cos\theta }{\sin\left(\frac{\theta }{swr}\right)}\;$$

(24b) $$\frac{d\theta }{dv_{p0}}=-\frac{\omega_{0}l}{v_{p0}^{2}}\;$$

(24c) $$\frac{dv_{p0}}{d\varepsilon_{MUT}}=-\frac{v_{p0}}{4\varepsilon_{eff}}\left(1-F\right)\;$$

(24d) $$\frac{d\phi }{dZ_{0}}=\frac{\frac{\omega_{0}C}{2}\sin\theta }{\sin\left(\frac{\theta }{swr}\right)}\;$$

(24e) $$\frac{dZ_{0}}{d\varepsilon_{MUT}}=-\frac{Z_{0}}{4\varepsilon_{eff}}\left(1-F\right)\;$$

where implementation in microstrip technology is considered. Introducing Equations (24) in Equation (23) gives:

(25) $$S=\frac{d\phi }{d\varepsilon_{MUT}}=\frac{1-F}{4\varepsilon_{eff}}\left\{\theta \cdot \frac{\sin\theta +\frac{\omega_{0}CZ_{0}}{2}\cos\theta }{\sin\left(\frac{\theta }{swr}\right)}-Z_{0}\cdot \frac{\frac{\omega_{0}C}{2}\sin\theta }{\sin\left(\frac{\theta }{swr}\right)}\right\}$$

It is very important to emphasize that for the calculation of the sensitivity, it is first necessary to set the reference *ε_MUT_*, that is, the value where the sensitivity is calculated. Such value is *ε_MUT_* = *ε_air_* = 1 in the present paper, but the reference dielectric constant can be set to a different value. The values of *Z*_0_, *C* and *θ* that result by inverting Equations (20)–(22) do not depend on the MUT on top of the sensing region. However, the geometry of the host line (length and width) depends on the reference *ε_MUT_*. In particular, the transverse geometry determines both the geometry factor, *F*, and the effective dielectric constant, *ε_eff_*, in (25). On the other hand, the interest is sensitivity optimization in the vicinity of the reference *ε_MUT_*. Naturally, *S*, as given by (25), depends on *θ*, *C* and *Z*_0_, but we must use (25) with some caution. That is, for sensor design, the characteristic impedance of the reactively-loaded structure should be preferably set to *Z_B_* = 50 Ω, since this is the reference impedance of the ports. Naturally, this impedance will be modified by the presence of the MUT in the sensing region, but, with such value, mismatching reflections will be minimized (especially for small perturbations), and the phase of the structure, *ϕ*, the output variable, will be similar to the phase of the transmission coefficient, an easily measurable quantity. Indeed, in [61], the effects of the variation in the dielectric constant of the MUT on the impedance of the host line, *Z*_0_, were neglected, but this only provides a rough estimate of the sensitivity. Indeed, by including such term, if the impedance of the structure is set to *Z_B_* = 50 Ω, it is not possible to infer a value of the sensitivity better than the one of an ordinary line with identical physical length and characteristic impedance, for reasonable values of the slow-wave factor. Nevertheless, by implementing the sensing line by means of a slow-wave structure based on shunt capacitors, the impedance of the host line increases (and the width decreases), thereby allowing meandering of the line, as depicted in Figure 5a, in a relatively small space. Therefore, the implementation of an ordinary line with the same impedance (and consequently with wider strip width) in the same sensing area does not easily allow the necessary length to achieve the same sensitivity (this has been experimentally corroborated in [61], to be discussed next). 

The slow-wave ratio of the structure was set to *swr* = 1/2 in [61]. This represents an appreciable reduction in sensor size and phase velocity, yet keeping an implementable value of the impedance of the host line, Z_0_. Thus, the unique parameter that should be determined is the phase of the unit cell, *ϕ*, related to the phase of the host line according to *θ = swr*⋅*ϕ*. For calculation of *S* by means of Equation (25) for different values of *ϕ*, and hence *θ*, it is necessary to obtain first Z_0_ and *C* using (20)–(22) for each value of *ϕ*, or *θ*, and then introduce the corresponding triplets (*θ*, Z_0_ and *C)* in (25). 

Inspection of (25) reveals that as the phase of the unit cell approaches *ϕ* = π, the sensitivity increases, but the impedance of the host line, *Z*_0_, increases, as well. Therefore, a tradeoff is necessary. Specifically, for *ϕ* = 120°, and the considered *swr* and *Z_B_*, inversion of Equations (20)–(22) gives Z_0_ = 150 Ω, *C* = 0.817 pF, and *θ* = 60^o^. This value of *Z*_0_ is achievable with many microwave substrates, but it is not convenient to deal with higher values of this parameter. For instance, by considering the commercial microwave substrate *Rogers RO4003C* with dielectric constant *ε_r_* = 3.55 and thickness *h* = 1.524 mm, and that the minimum strip width achievable with the fabrication technology in use is 200 μm, the maximum implementable characteristic impedance of the host line (considering that it is surrounded by air) is *Z*_0,max_ = 167 Ω.

**Figure 6:**
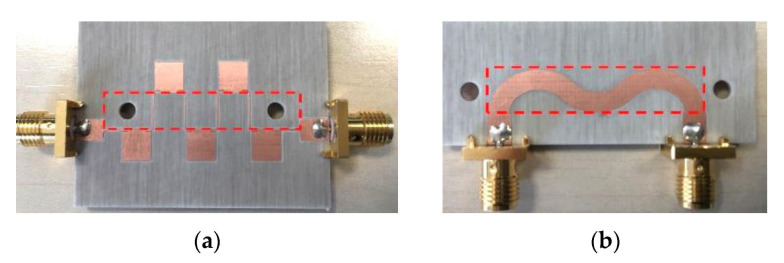
Photograph of the fabricated sensors. (**a**) Capacitively-loaded line sensor; (**b**) meander line sensor. Dimensions (in mm) are: *l_C_* = 5.0, *l_acc_* = 0.2, *W_acc_* = 3.0, *l_h_*_,1_ = 3.19, *l_h_*_,2_ = 5.35, and *W_h_* = 0.23 [in reference to Figure 5a]. Reprinted with permission from [61].

**Figure 7:**
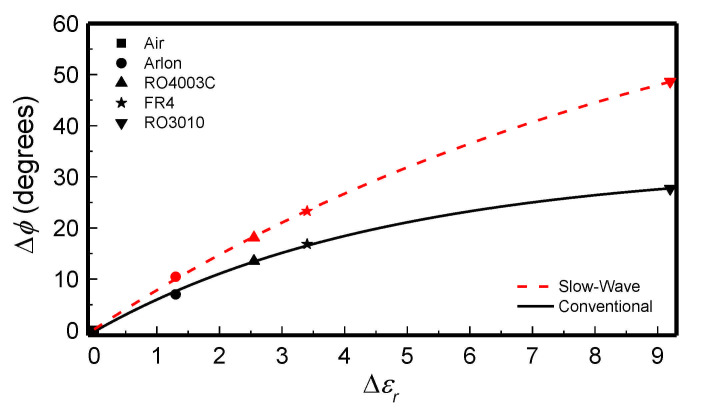
Dependence of the differential phase of the measured transmission coefficient at the operating frequency with the dielectric constant of the MUT. The differential phase is defined as Δ*ϕ* = *N*⋅*ϕ_MUT_* *− N*⋅*ϕ_air_*, where *N*⋅*ϕ_MUT_* and *N*⋅*ϕ_air_* are the measured phases obtained with MUT and air, respectively, and Δ*ε_r_* = *ε_r_*_,*MUT*_ − *ε_r_*_,*air*_. Reprinted with permission from [61].

With the previously indicated line parameters (Z_0_ = 150 Ω, *C* = 0.817 pF, and *θ* = 60^o^) and substrate, the sensing structure (unit cell) of Figure 5a was obtained (the operating frequency was set to *f*_0_ = 3 GHz). The sensor was fabricated by considering *N* = 5 unit cells. Meandering in the host line was applied in order to obtain a sensing area (indicated with the dashed rectangle) with a reasonable shape factor. The photograph of the sensor (fabricated by means of the *LPKF H100* drilling machine) is depicted in Figure 6a. To experimentally validate the improvement in the sensitivity, as compared to the one achieved with an ordinary line, a 50 Ω meandered line sensor with roughly the same sensing area was also fabricated (Figure 6b). By loading the sensing area of both sensors with materials of different dielectric constants, the measured differential phase of the transmission coefficient, for *f*_0_ = 3 GHz, as a function of the dielectric constant of the MUT was obtained (Figure 7). As it can be seen, the phase experiences a stronger variation with the dielectric constant for the slow-wave based sensor.

In the reported example, the host line constitutes the sensing region, and the changes in the phase and characteristic impedance of such line, caused by the presence of the MUT on top of it, are responsible for the variation in the phase of the reactively-loaded line, the output variable. An alternative approach, where the sensing region is formed by the loading patch capacitors, has been recently reported [90]. In this case, the working principle is the variation of the coupling (edge) capacitance between adjacent patches, which must be closely spaced. The sensitivity is also significantly better than that of the ordinary meander line with similar sensing area. The advantage of this later approach concerns the fact that the sensing region can be clearly separated from the host line, thereby easing the design. By contrast, separation of the sensing region (the host line) in the structure of Figure 6a from the rest of the reactively loaded line is not so simple. Nevertheless, both approaches have been demonstrated to be useful for sensitivity enhancement in transmission-mode phase-variation sensors. We would also like to mention that reactively-loaded slow-wave transmission lines can be implemented by means of series inductors. In practice, such inductively-loaded lines can be implemented by means of microstrip lines periodic loaded with dumbbell-shaped defect ground structure (DB-DGS) resonators operating below resonance (where they exhibit a positive, i.e., inductive, reactance). In this case, since the loading elements are slot resonant elements placed in the ground plane, the sensing region (host line) is completely separated from the loading elements, thereby easing the design. Work is in progress towards the implementation of phase-variation transmission-mode sensors based on such inductively loaded lines.

## 5. Phase-Variation Sensors Based on Step-Impedance Line Sections and Open-Ended Quarter- and Half-Wavelength Sensing Lines

Contrary to the phase-variation sensors reviewed in the previous two sections, operating in transmission, the operational mode of the sensors subject of this section is reflection. Specifically, the considered devices are one-port reflective-mode phase-variation sensors based on an open-ended sensing line cascaded to a step-impedance transmission line. Such sensors, first reported in [62], can potentially exhibit very huge sensitivities without the need of increasing the size of the sensing region. The key aspect for sensitivity enhancement in such sensors is the presence of the step-impedance transmission line. As it will be shown, the sensitivity, or variation of the phase of the reflection coefficient (the output variable) with the dielectric constant of the MUT (the input variable), is enhanced by the impedance contrast of the high/low-impedance transmission line sections of the step-impedance configuration. 

**Figure 8:**
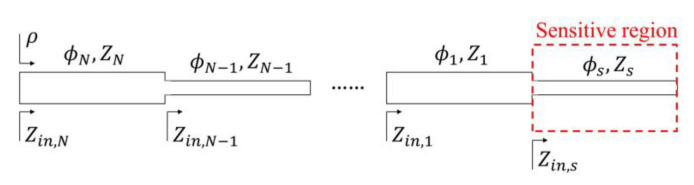
Schematic of the reflective-mode phase-variation sensor based on an open-ended sensing line cascaded to a step-impedance transmission line structure. Reprinted with permission from [62].

The typical schematic of these reflective-mode phase-variation sensors is depicted in Figure 8. The open-ended sensing line is described by its electrical length *ϕ_s_* and characteristic impedance *Z_s_*. The variables *ϕ_i_* and *Z_i_* are used to designate the phase and characteristic impedance, respectively, of the different high/low-impedance transmission line sections, differentiated by the index *i*, with *i* = 1, 2, 3…*N*, where *N* is the number of line sections excluding the sensing line. The impedance seen from the different planes of the structure (corresponding to the step-impedance discontinuities), looking at the open-end, are designated by the variable *Z_in,i_*, and *Z_in,s_* is the impedance seen from the plane of the sensing line opposite to the open-end. It was demonstrated in [62] that for sensitivity optimization, the sensing line must be either a high-impedance 90° line [or, more generally a (2*n* + 1)⋅π/2 line], or a low-impedance 180^o^ line (or, more generally, a *n*⋅π line), where the phase of such line should take these alternative values at the operating frequency, *f*_0_. Moreover, the step-impedance structure must be designed with 90° line sections of alternative high/low impedance, where the impedance of the first section (*i* = 1), the one adjacent to the sensing line, must be high and low for the 180^o^ and 90^o^ sensing lines, respectively. 

As reported in [62], the impedance seen from the input port is:

(26) $$Z_{in,N}={\left(-jZ_{s}\right)}^{{\left(-1\right)}^{N}}\cdot {\left(\cot\phi_{s}\right)}^{{\left(-1\right)}^{N}}\cdot \overset{N}{\underset{i=1}{\prod }}\left\{Z_{i}^{2\cdot {\left(-1\right)}^{i+N}}\right\}\;$$

where the symbol Π denotes the product operator. The reflection coefficient seen from the input port is thus:

(27) $$p=\frac{j{\left(-1\right)}^{N+1}\cdot {\left(Z_{s}\cot\phi_{s}\right)}^{{\left(-1\right)}^{N}}\cdot \prod_{i=1}^{N}\left\{Z_{i}^{2\cdot {\left(-1\right)}^{i+N}}\right\}-Z_{0}}{j{\left(-1\right)}^{N+1}\cdot {\left(Z_{s}\cot\phi_{s}\right)}^{{\left(-1\right)}^{N}}\cdot \prod_{i=1}^{N}\left\{Z_{i}^{2\cdot {\left(-1\right)}^{i+N}}\right\}+Z_{0}}\;$$

and the phase of the reflection coefficient, the output variable is given by:

(28) $$\phi_{p}=2\arctan\left(\frac{{\left(-1\right)}^{N}\cdot {\left(Z_{s}\cot\phi_{s}\right)}^{{\left(-1\right)}^{N}}\cdot \prod_{i=1}^{N}\left\{Z_{i}^{2\cdot {\left(-1\right)}^{i+N}}\right\}}{Z_{0}}\right)$$

The sensitivity can be expressed as [62,91]:

(29) $$S=\frac{d\phi_{\rho }}{d\varepsilon_{MUT}}=\frac{d\phi_{\rho }}{d\phi_{s}}\cdot \frac{d\phi_{s}}{d\varepsilon_{MUT}}+\frac{d\phi_{\rho }}{dZ_{s}}\cdot \frac{dZ_{s}}{d\varepsilon_{MUT}}\;$$

However, as discussed in [62,91], the last term in the right-hand side member is null for the optimum phase of the sensing line, either 90° or 180°, provided *dϕ_ρ_*/*dZ_s_* = 0 for those phases. Concerning the second derivative of the first term, it is given by:

(30) $$\frac{d\phi_{s}}{d\varepsilon_{MUT}}=\;\frac{\phi_{s}}{4\varepsilon_{eff}}\left(1-F\right)\;$$

where implementation in microstrip technology is considered. For the first derivative, it is found to be:

(31) $$S_{\phi_{s}}\equiv \frac{d\phi_{\rho }}{d\phi_{s}}=-\frac{2}{\frac{Z_{0}}{\prod_{i=1}^{N}\left\{Z_{i}^{2\cdot {\left(-1\right)}^{i+N}}\right\}\cdot {\left(Z_{s}\right)}^{{\left(-1\right)}^{N}}}\cdot \frac{{\left(\sin\phi_{s}\right)}^{{\left(-1\right)}^{N}+1}}{{\left(\cos\phi_{s}\right)}^{{\left(-1\right)}^{N}-1}}\;+\;\frac{\prod_{i=1}^{N}\left\{Z_{i}^{2\cdot {\left(-1\right)}^{i+N}}\right\}\cdot {\left(Z_{s}\right)}^{{\left(-1\right)}^{N}}}{Z_{0}}\;\cdot \frac{{\left(\cos\phi_{s}\right)}^{{\left(-1\right)}^{N}+1}}{{\left(\sin\phi_{s}\right)}^{{\left(-1\right)}^{N}-1}}}\;$$

In order to evaluate (31) for the phases of interest, *ϕ_s_* = 90° or *ϕ_s_* = 180°, it is necessary to distinguish if the number of sections, *N*, is even or odd. Hence, four different cases appear. The sensitivity for each case is found to be:

Case A: *ϕ_s_* = (2*n* + 1)⋅π/2 and *N* odd:

(32a) $$S_{\phi_{s}}=-\frac{2Z_{s}Z_{0}}{\prod_{i=1}^{N}\left\{Z_{i}^{2\cdot {\left(-1\right)}^{i+N}}\right\}}\;$$

Case B: *ϕ_s_* = *n*⋅π and *N* odd:

(32b) $$S_{\phi_{s}}=-\frac{2\cdot \prod_{i=1}^{N}\left\{Z_{i}^{2\cdot {\left(-1\right)}^{i+N}}\right\}}{Z_{s}Z_{0}}\;$$

Case C: *ϕ_s_* = (2*n* + 1)⋅π/2 and *N* even:

(32c) $$S_{\phi_{s}}=-\frac{2Z_{s}\cdot \prod_{i=1}^{N}\left\{Z_{i}^{2\cdot {\left(-1\right)}^{i+N}}\right\}}{Z_{0}}\;$$

Case D: *ϕ_s_* = *n*⋅π and *N* even:

(32d) $$S_{\phi_{s}}=-\frac{2Z_{0}}{Z_{s}\cdot \prod_{i=1}^{N}\left\{Z_{i}^{2\cdot {\left(-1\right)}^{i+N}}\right\}}\;$$

Note that *Z_s_* appears in the numerator for *ϕ_s_* = (2*n* + 1)⋅π/2 and it appears in the denominator for *ϕ_s_* = *n*⋅π. It is clear from this result that for quarter-wavelength (or odd multiple) sensing lines, a high characteristic impedance is needed for sensitivity optimization (regardless of the number of sections of the structure). Conversely, for half-wavelength (or multiple) sensing lines, the characteristic impedance must be low. From the analysis of the product operator that appears in Equations (32) we can conclude that:

(i) For *N* odd (cases A and B), the characteristic impedance of a section with odd order (*i* odd) appears as $Z_{i}^{2}$. By contrast, for an even-order section, the corresponding term in the product appears as the inverse, i.e., $Z_{i}^{-2}$. According to this, the requirement of a high or low value of *Z_i_* for sensitivity optimization depends on whether the product operator is present either in the numerator or in the denominator in Equations (32). For case A, where the product operator appears in the denominator, the odd-order transmission line sections must exhibit low impedance values, whereas high characteristic impedance sections are required for the even sections. For case B, the opposite conditions apply, since the product operator appears in the numerator of (32b).
(ii) For *N* even (cases C and D) and *i* odd, the impedance is negative squared ($Z_{i}^{-2}$), whereas it appears as $Z_{i}^{2}$ for *N* even and *i* even. This means that for case C, with the product operator in the numerator of (32c), the odd sections must exhibit low characteristic impedance, and the line impedance must be high for the even sections. It is obvious that for case D (half-wavelength sensing line), the sections that should exhibit high impedance for sensitivity optimization are those with odd index.

According to the previous explanations, the need for a step-impedance configuration, including the sensing line, in order to maximize the sensitivity as much as possible, is clear. It should be emphasized that the sensitivity that results by introducing Equations (32) and (30) in Equation (29), is the one corresponding to the indicated phases at the operating frequency. Naturally, these phases are invariant for the high/low-impedance 90^o^ line sections enumerated by the index *i*, regardless of the dielectric constant of the MUT on top of the sensing line, if it is present. However, it is obvious that for the sensing line, the phase depends on *ε_MUT_*. Therefore, the phase requirement for sensitivity optimization, either *ϕ_s_* = 90° or *ϕ_s_* = 180°, depends on the value of *ε_MUT_*. If such value is *ε_MUT_*= 1, the dielectric constant of air, this means that the sensitivity is optimized for variations of the dielectric constant in the vicinity of that of air. This was the case considered in [62]. However, it has been recently demonstrated that the sensitivity can be efficiently optimized for other (arbitrary) values of the dielectric constant of the MUT [64]. Obviously, when the sensitivity is evaluated, it is important to bear in mind that the effective dielectric constant in (30), *ε_eff_*, is the one given by (4) with the *ε_MUT_* value corresponding to the reference MUT (i.e., the one providing the required optimum phase for the sensing line).

**Figure 9:**
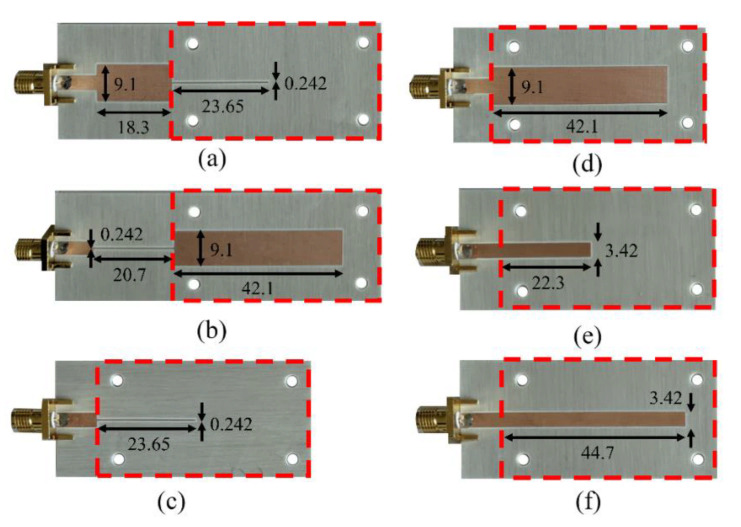
Photographs of the fabricated reflective-mode phase-variation sensors. (**a**) Sensor with step-impedance discontinuity, *Z*_1_ < *Z*_0_ < *Z_s_*, and *ϕ_s_* = 90°; (**b**) sensor with step-impedance discontinuity and *Z*_1_ > *Z*_0_ > *Z_s_*, and *ϕ_s_* = 180°; (**c**) sensor with uniform mismatched *ϕ_s_* = 90° sensing line and *Z_s_* > *Z*_0_; (**d**) sensor with uniform mismatched *ϕ_s_* = 180° sensing line and *Z_s_* < *Z*_0_; (**e**) sensor based on a 90° uniform 50 Ω sensing line and (**f**) sensor based on a 180° uniform 50 Ω sensing line. The considered high and low impedances are 150 Ω and 25 Ω, respectively. The dimensions are given in the pictures (in mm), and the sensing regions are indicated by dashed rectangles. Reprinted with permission from [62].

To illustrate the approach discussed in this section, we report the phase variation of the reflection coefficient, as a function of the dielectric constant of the MUT, for six different sensors, depicted in Figure 9. All these sensors are implemented in microstrip technology, and the reference MUT is air. That is, the phase of the sensing line at the operating frequency (*f*_0_ = 2 GHz) is 90° or 180° when the sensing line is uncovered. The sensors were implemented on the Rogers RO4003C substrate with dielectric constant *ε_r_* = 3.55, thickness *h* = 1.524 mm and loss factor tanδ = 0.0022 (the prototypes were fabricated by means of the LPKF H100 drilling machine. 

**Figure 10:**
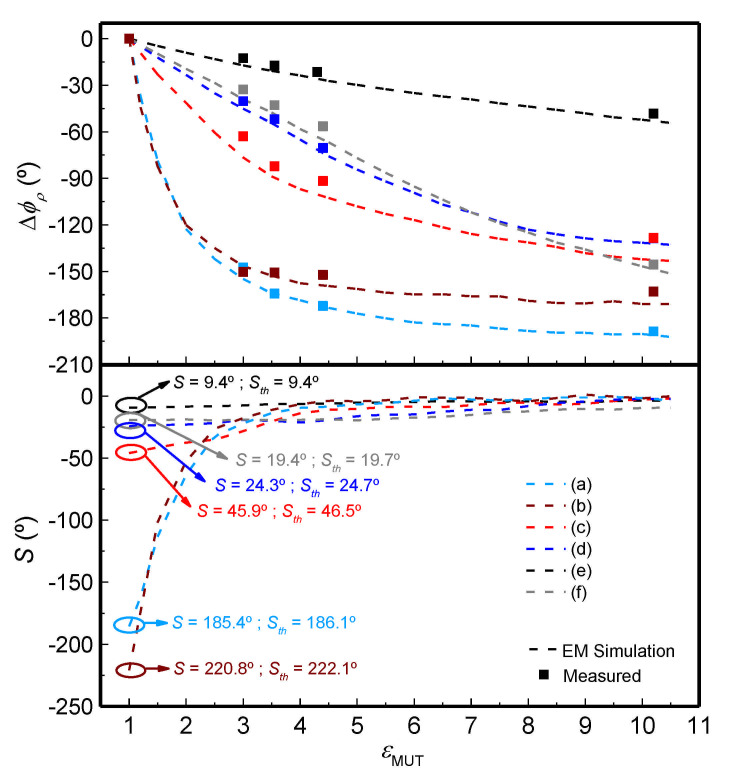
Measured and simulated phase of the reflection coefficient for the sensors of Figure 9, and simulated sensitivity. (**a**) Sensor with step-impedance discontinuity and *Z* < *Z*_0_ < *Z_s_*; (**b**) sensor with step-impedance discontinuity and *Z* > *Z*_0_ > *Z_s_*; (**c**) sensor with uniform mismatched sensing line and *Z_s_* > *Z*_0_; (**d**) sensor with uniform mismatched sensing line and *Z_s_* < *Z*_0_; (**e**) sensor based on a 90° uniform 50 Ω sensing line and (**f**), sensor based on a 180^o^ uniform 50 Ω sensing line. The measured dielectric loads are 3 mm slabs of uncoated *PLA* (*ɛ_MUT_* = 3), Rogers RO4003C (*ɛ_MUT_* = 3.55), *FR4* (*ɛ_MUT_* = 4.4) and Rogers RO3010 (*ɛ_MUT_* = 10.2) substrates. The sensitivities in the limit of small perturbations are given in absolute value. Reprinted with permission from [62].

Note that sensors A and B consist of the sensing line plus a single 90° line section, whereas sensors C, D, E and F are implemented solely with the sensing line. The simulated phase for the different sensors as a function of the dielectric constant of the MUT, semi-infinite in the vertical direction, is depicted in Figure 10. The figure also includes the measured phase for different MUT samples, as well as the sensitivity, inferred from the simulated data points (the number of experimental data points is very limited). The sensitivities for small perturbations are in good agreement with the values predicted by theory, indicated as *S_th_* in the figure. It is remarkable that the sensitivities for sensors A and B are very high (especially for sensor B), by virtue of the step-impedance configuration.

The sensitivity can be further enhanced by merely cascading additional 90° line sections to sensors A and B of Figure 7. In [62], a sensor with *N* = 2 quarter wavelength (90°) stages, based on a 90° sensing line, and impedances set to *Z_s_* = 150 Ω, *Z*_1_ = 25 Ω and *Z*_2_ = 86.6 Ω, was also reported. The achieved sensitivity for small perturbations (i.e., in the limit were *ε_MUT_* = 1) was as high as 528.7°, a very competitive value. With such sensitivity, the *FoM*, defined as the ratio between the maximum sensitivity and the area of the sensing region expressed in terms of the squared guided wavelength, is found to be *FoM* = 21148° /λ^2^. However, linearity is poor in this sensor, specifically devoted to measure tiny variations in the dielectric constant in the vicinity of the reference dielectric constant, i.e., the one of air. For this reason, experimental data concerning the MUT samples of Figure 10 were not considered for this sensor. 

In the sensors of Figure 9, the reference MUT is air, which means, as indicated before, that sensitivity is optimized for variations of the dielectric constant in the vicinity of that of air. In a recent paper [64], it was shown that the sensitivity can be optimized for different values of the reference dielectric constant. Particularly, the reference value was considered to be *ε_MUT_* = 3.55 in the sensor reported in [64]. It was also shown in that paper that these sensors are able to detect tiny defects in samples, provided such defects alter the dielectric constant of the MUT. For example, drilling an array of holes in the sample modifies the effective dielectric constant of such sample. Thus, it is expected that by loading the sensitive region (sensing line) with samples with different densities of holes, those samples exhibiting a larger density experience a stronger variation of the phase of the reflection coefficient. This functionality as defect detector was demonstrated in [64]. 

**Figure 11:**
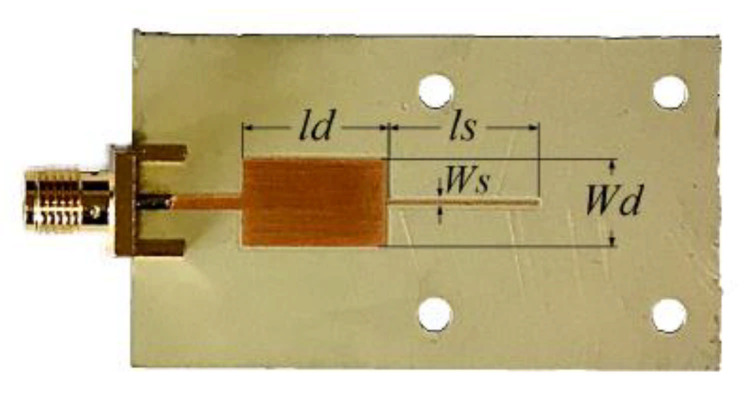
Photograph of the designed and fabricated reflective-mode phase-variation sensor based on a low-impedance 90° line section and a high-impedance 90° open-ended sensing, with reference sample the uncoated Rogers 4003C substrate (with *ε_MUT_* = 3.55). Dimensions (in mm) are: *W_d_* = 7.5, *l_d_* = 12.8, *W_S_* = 0.235 and *l_S_* = 13.5. Reprinted with permission from [64].

For illustration purposes, let us report a sensor structure consisting of a 90° high-impedance open-ended sensing cascaded to a 90° low-impedance line section [64]. The reference MUT is an uncladded Rogers 4003C slab with *ε_MUT_* = 3.55. The photograph of the sensor is depicted in Figure 11, where the dimensions are indicated. The considered sensor substrate was the Rogers RO3010 with dielectric constant *ε_r_* = 10.2, thickness *h* = 1.27 mm and loss tangent tanδ = 0.0023, and the operating frequency was set to *f*_0_ = 2 GHz. The high and low impedance values of the sensing line and cascaded line are *Z_s_* = 85 Ω and *Z*_1_ = 15 Ω, respectively.

**Figure 12:**
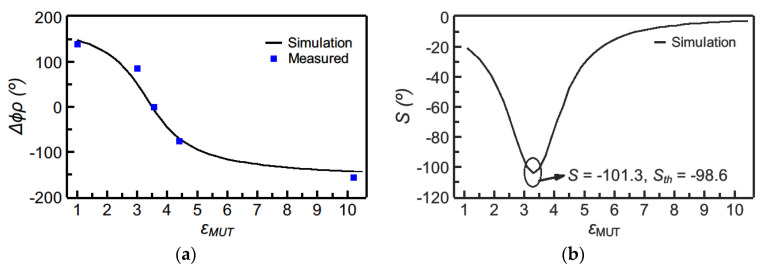
Dependence of the differential phase of the reflection coefficient (i.e., Δ*ϕ_ρ_* = *ϕ_ρ_* − *ϕ_ρ_*_,*REF*_) with the dielectric constant of the MUT (**a**) and sensitivity (**b**), for the sensor of Figure 11. The measured data points have been obtained by covering the sensing line with the following uncoated materials: Rogers RO3010 (*ε_MUT_* = 10.2), *FR4* (*ε_MUT_* = 4.4), Rogers RO4003C (*ε_MUT_* = *ε_REF_* = 3.55), *PLA* (*ε_MUT_* = 3.0, and fabricated by means of a 3D printer). Additionally, the phase of the reflection coefficient has been experimentally obtained with the sensing line uncovered (*ε_MUT_*_, air_ = 1). Reprinted with permission from [64].

**Figure 13:**
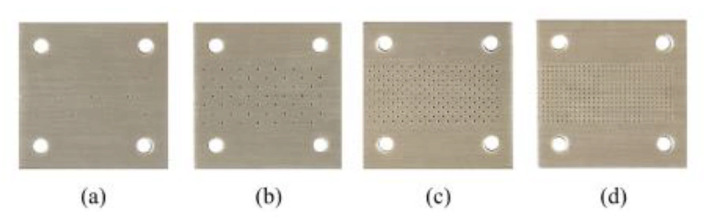
Reference sample with arrays of holes of different densities drilled across the substrate. From sample (**a**) to sample (**d**), the density of holes increases. Reprinted with permission from [64].

**Figure 14:**
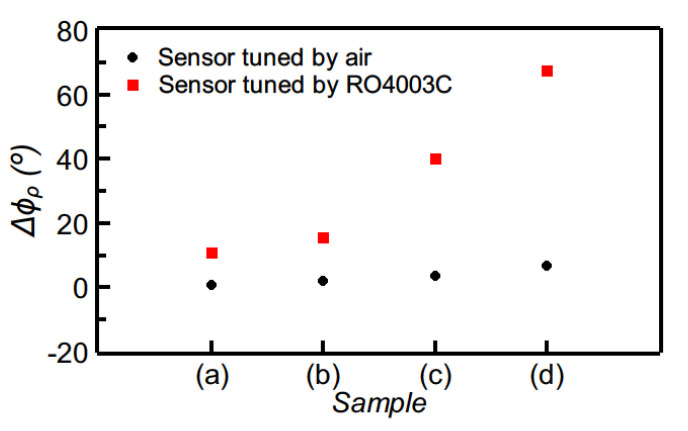
Comparison of the measured differential phase of the reflection coefficient for the sensor of Figure 11 and for the equivalent sensor, but with the sensing line exhibiting a phase of 90° when it is uncovered (i.e., with air as reference material). Reprinted with permission from [64].

The phase variation as a function of the dielectric constant of the MUT sample, inferred from electromagnetic simulation, as well as the resulting sensitivity are depicted in Figure 12 (the figure includes several experimental data points, corresponding to the MUT samples of Figure 10). As it can be appreciated, the maximum sensitivity, indicated in the figure, is very high. For defect detection, four samples of the reference material were modified by drilling arrays of holes with different densities as depicted in Figure 13. The phase variation for each sample is depicted in Figure 14. The sensor is able to perfectly distinguish the different samples, thanks to its high sensitivity in the vicinity of the dielectric constant of the reference sample. However, if the same sensor is designed by considering air as reference sample, the device does not exhibit such excellent capability to differentiate among the different defected samples.

## 6. Other Phase-Variation Sensors

In this section, two phase-variation sensors are reported. In one case, the sensor is devoted to dielectric characterization, but the phase information is translated to magnitude information. In the second case, a sensor devoted to rotation measurements is reviewed, in order to show the potential of sensing other variables based on phase measurements.

### 6.1. Differential Phase-Variation Sensors Based on a Composite Right/Left Handed (CRLH) Line

**Figure 15:**
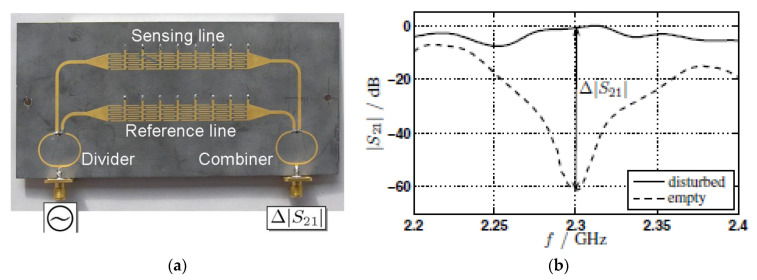
Photograph of the CRLH transmission line based differential-mode phase variation sensor (**a**) and comparison between the transmission coefficients for the empty case (unloaded sensing line) and for the sensing line loaded with a MUT (**b**). Reprinted with permission from [38].

In [38], a differential-mode sensor based on a pair of CRLH lines was reported. Such lines are very dispersive [70,72], and therefore the phase is very sensitive to the presence of a material on top of them. The photograph of the sensor reported in [38] is depicted in Figure 15. The pair of CRLH lines are implemented by means of a host microstrip line with series interdigital capacitors and shunt-connected inductive strips (grounded by means of vias). If the loading of both lines is different, e.g., one of them is surrounded by air, and the other one by means of a certain material (MUT), the dielectric properties (dielectric constant) of such material modifies the phase of the corresponding line. Consequently, the differential phase, the typical output variable in such differential-mode sensors, takes a value that depends on the difference of the dielectric constant between the MUT and the REF material, similar to the differential-mode phase-variation sensor based on meandered lines and reported in Section 3. However, in the device shown in Figure 15, it can be appreciated that a power splitter is connected to the differential input port, whereas a combiner is connected to the differential output port. However, the feeding lines of the upper CRLH line are one half wavelength longer than the ones for the lower CRLH line, so that the signals at the end of both lines show a phase difference of 180°. Thus, if the lines are identical, a destructive interference is caused at the output port of the combiner. However, if the electrical length of one of the two parallel lines is changed, the interference is not completely destructive anymore and the output signal increases.

Obviously, the CRLH lines can be designed such that the interference is completely constructive for certain line imbalance, corresponding to the required input dynamic range. Figure 15b depicts the frequency response of the differential-mode phase-variation sensor for two cases, i.e., with unloaded (empty) sensing line, and with the sensing line loaded with a MUT (disturbed case). The combination of CRLH line characteristics and *ε_MUT_* was determined in [38] in order to obtain the maximum possible transmission coefficient when the MUT is on top of the sensing line (this occurs provided the signals at the input ports of the combiner are in-phase). The output dynamic range at the operating frequency, *f*_0_ = 2.3 GHz, is roughly 60 dB. Further details on this structure can be found in [38].

### 6.2. Phase-Variation Sensor for Rotation Measurements

**Figure 16:**
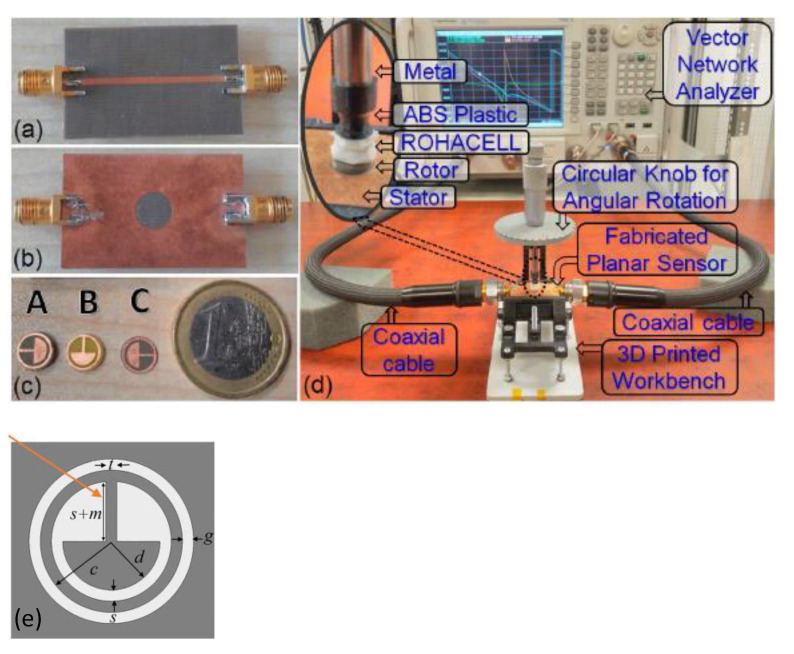
Photographs of the phase-variation sensor system based on a microstrip line loaded with a rotatable CSRR. (**a**) Bottom and (**b**) top views of the stator, fabricated on the 0.5-mm-thick *RT5880* substrate with dielectric constant *ε_r_* = 2.2*,* and dissipation factor tanδ = 0.009; (**c**) rotor A (1.5-mm-thick *RT5880* laminate, *ε_r_* = 2.2*,* tanδ = 0.009), rotor B (1-mm-thick *FR4* laminate, *ε_r_* = 4.3, tanδ = 0.025), and rotor C (0.5-mm-thick RT5880 laminate); (**d**) measurement setup for directional angular rotation. The microstrip line length and width are 40 mm and 1.49 mm. The dimensions of the MCSRR, in regard to the (**e**) zoom view topology, are: *c* = 3.5 mm, *d* = 1.5 mm, *s* = 0.5 mm, *t* = 0.5 mm, *g* = 0.5 mm. Reprinted with permission from [65].

**Figure 17:**
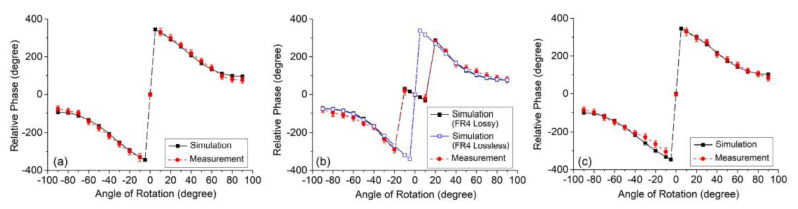
Differential phase of the reflection coefficients seen from the ports for the sensor of Figure 16, for (**a**) rotor A, (**b**) rotor B, and (**c**) rotor C. Reprinted with permission from [65].

Several phase-variation sensors devoted to the measurement of angular or linear displacements have been reported in the literature [65,66,67]. In this paper, one of such sensors is succinctly reviewed. It consists of a rotary sensor based on a microstrip line loaded with a movable complementary split ring resonator (CSRR) [65], see Figure 16. The output variable of this sensor is the difference in the phase of the reflection coefficients seen from both ports. Note that the considered CSRRs exhibit a symmetry plane. Thus, if their orientation with regard to the line is with such symmetry plane orthogonal to the line axis, perfect symmetry in the mid plane between the ports arises, and the differential output signal is null. However, by rotating the CSRR, such symmetry is disrupted, and the phase of the reflection coefficients are no longer identical. Such phase difference is enhanced by cross-polarization of the CSRRs, i.e., by mixed coupling (electric and magnetic) between the line and the CSRR when it is rotated. The fabricated stator and rotor are depicted in Figure 16 (actually, sensor functionality by considering several rotors, as depicted in the figure, was demonstrated in [65]). Note that the considered resonators of the rotors exhibit a modified topology as compared to the circular topology usually considered. This modified CSRR (MCSRR) was used in [65] in order to enhance the magnetic coupling with the line, thereby enhancing the cross-polarization effects, with direct impact on sensitivity optimization. Figure 17 depicts the phase response of the sensor with the rotation angle for the different considered rotors.

The maximum sensitivities achieved with rotors A, B and C (see Figure 16) are 3.2, 2.9 and 3.1, respectively. It is worth mentioning that the sign of the differential phase (the output variable) indicates if the angular displacement with regard to the reference angular position (symmetric orientation of the rotor) proceeds clockwise or counterclockwise (see Figure 17).

## 7. Comparative Analysis

For comparison purposes, Table 1 includes several phase-variation microwave sensors devoted to dielectric constant measurements and their main relevant characteristics. The operation mode (reflection or transmission), the size of the sensing area, the maximum sensitivity, and the main Figure of Merit (*FoM*), defined as the ratio between the maximum sensitivity and the sensing area expressed in terms of the squared guided wavelength, are indicated in the table. Moreover, the sensors are grouped according to the three categories corresponding to Section 3, Section 4 and Section 5 of this paper, i.e., meander-line based sensors, artificial transmission-line based sensors, and sensors based on impedance contrast (i.e., using a step-impedance transmission line configuration). 

Though the sensing principle of the devices reported in Table 1 is phase variation, the output variable is not necessarily a phase. That is, phase-to-magnitude conversion is possible, and, in this case, the output variable is typically the magnitude of a transmission coefficient [38,45,46]. Thus, for example, in the sensor reported in [25], working differentially, a pair of meandered lines is used, but the differential phase is transformed to magnitude by adding a pair of rat-race hybrid couplers to the sensitive part of the device. The resulting structure is a two-port sensor, and the output variable is the magnitude of the transmission coefficient. The sensitivity of such sensors is very good, as it can be appreciated in the table, but the area of the whole structure is significant due to the presence of the two rat race couplers. In [38] and [45], the sensors exploit dispersion engineering in composite right/left-handed (CRLH) lines and electro-inductive-wave (EIW) transmission lines, respectively. In such sensors the phase information is also converted to magnitude information, and, for this reason, the sensitivity is expressed in dB. These sensors exhibit very good sensitivity but the design of the artificial lines (either CRLH or EIW lines) is not simple. This also applies to the sensor based on slow-wave transmission lines reported in [61], and reviewed in Section 4.

Inspection of those sensors in Table 1 where the output variable is a phase (so that the sensitivity is expressed in degrees) reveals that the *FoM* is excellent in the reflective-mode sensors reported in [62]. The reason is that a high sensitivity is achievable in such sensors without the need to modify the sensing region, either a 90° or a 180° open-ended line. By contrast, in the sensors reported in [37], based on meandered lines, the sensitivity is also very good, but such high sensitivity is achieved at the expense of increasing the length of the sensing line. For this reason, the *FoM* is not as good as the one of the reflective mode sensors reported in [62]. Indeed, the main relevant and unique feature of the step-impedance transmission line based sensors of Section 5 (a subset of those reported in [62,63,64]) is the fact that the sensitivity can be enhanced at wish by merely adding high/low impedance 90° transmission line sections. Moreover, the design of these sensors is very simple, as far as they are based on a cascade of transmission line sections. Besides these advantageous aspects, these sensors operate in reflective-mode and at a single frequency. With all these characteristics and attributes, it is believed that these sensors may find practical application, in applications requiring very high sensitivity. Examples could be defect detection, measurement of electrolyte content in bio-samples (e.g., blood or urine), or determination of alcohol content in wine fermentation processes, among others. For applications to the characterization of liquids, adding microfluidic channels to the sensing region is an approach, but, alternatively, it is also possible to consider submersible sensors, as far as they operate in reflection.

As mentioned, phase-variation sensors are especially suited to measure dielectric constants and magnitudes related to it, such as material composition, defects, etc. The reason is that the phase of transmission lines is very sensitive to the effects of the dielectric constant of the surrounding medium. In some cases, measurement of the loss tangent has been reported, see Figure 4 [37], but in general, resonant methods tend to be more accurate for that purpose. 

## 8. Conclusions

In conclusion, several approaches for the implementation of phase-variation microwave sensors devoted to the dielectric characterization of materials have been reviewed in this paper. The main aim of the paper has been the discussion about specific strategies for sensitivity optimization, which include line elongation through meandering, dispersion engineering, and impedance contrast. Prototype examples representative of the considered approaches have been reviewed, in particular a differential transmission-mode meandered line sensor, a transmission-mode sensor based on a slow-wave transmission line, and, finally, a reflective-mode sensor based on an open-ended step-impedance transmission line configuration. Contrarily to the meandered-line and slow-wave based sensors, in the reflective-mode sensors discussed in the paper, the sensitivity can be enhanced by keeping the area of the sensing region unaltered. This is a key aspect of these reflective-mode step-impedance based sensors, and a unique feature, which allows boosting up the main figure of merit of phase-variation sensors, i.e., the ratio between the sensitivity and the area of the sensing region expressed in terms of the square wavelength. The value of the *FoM* in one of the reported reflective-mode sensors is as high as *FoM* = 2208°/*λ*^2^ (sensor B in Section 5), a very competitive value. However, this value has been even enhanced, as reported in Table 1, with a *FoM* = 21148°/*λ*^2^ achieved in a reflective mode sensor based on two 90° high/low-impedance line sections cascaded to the 90° sensing line (nevertheless, the linearity is very limited in this sensor, and variations of dielectric constant beyond *ε_MUT_* = 2, the reference being *ε_REF_* = 1, cannot be detected). In summary, the reported phase-variation sensors exhibit very good performance (specifically the sensitivity), size (sensing region), and cost. Moreover, these sensors operate at a single frequency and are robust against electromagnetic interference and noise. With all these characteristics, the reviewed sensors may find application in various scenarios, including industrial processes and medical diagnosis and monitoring, among others. 

## Figures and Tables

**Table 1:** Comparison of various phase variation-sensors.

Ref.	Type	Mode	Size * (λ^2^)	Max. Sensitivity	*FoM* (°/*λ*^2^)
[62]	IMPEDANCE CONTRAST	REFLECTIVE	0.025	528.7°	21148
[63]	IMPEDANCE CONTRAST	REFLECTIVE	0.1	45.5°	455
[64]	IMPEDANCE CONTRAST	REFLECTIVE	0.025	101.3°	4052
[38]	ARTIFICIAL LINE (CRLH)	TRANSMISSION	---	600 dB	---
[36]	MEANDER LINE	TRANSMISSION	---	54.8°	---
[37]	MEANDER LINE	TRANSMISSION	12.9	415.6°	32.2
[45]	ARTIFICIAL LINE (EIW)	TRANSMISSION	0.075	25.3 dB	---
[46]	MEANDER LINE	TRANSMISSION	0.02	17.6 dB	---
[61]	ARTIFICIAL LINE (SLOW-WAVE)	TRANSMISSION	0.03	7.7°	257
[90]	ARTIFICIAL LINE (SLOW-WAVE)	TRANSMISSION	0.04	20.0°	500

* The size corresponds to the sensing region, not to the whole sensing structure. In [62,63,64], the size has been calculated by multiplying the length of the sensing line (0.25λ or 0.50λ) by either 0.1λ or 0.2λ, the latter applied to CPW sensing lines or to low-impedance 180^o^ microstrip sensing lines (both cases corresponding to lines with a non-negligible width). Note that the dashed rectangles in the sensors of Figure 9 provide the dimensions of the MUT samples, by far superior to the area that is actually needed for measurement purposes, determined according to the indicated procedure.

## Data Availability

Not applicable.
